# Interaction Pattern of Arg 62 in the A-Pocket of Differentially Disease-Associated HLA-B27 Subtypes Suggests Distinct TCR Binding Modes

**DOI:** 10.1371/journal.pone.0032865

**Published:** 2012-03-05

**Authors:** Elisa Nurzia, Daniele Narzi, Alberto Cauli, Alessandro Mathieu, Valentina Tedeschi, Silvana Caristi, Rosa Sorrentino, Rainer A. Böckmann, Maria Teresa Fiorillo

**Affiliations:** 1 Department of Biology and Biotechnology “C. Darwin”, Sapienza University, Rome, Italy; 2 Computational Biology, Department of Biology, University of Erlangen-Nürnberg, Erlangen, Germany; 3 2nd Chair of Rheumatology, Department of Medical Sciences, University of Cagliari, Cagliari, Italy; 4 Istituto Pasteur-Fondazione Cenci Bolognetti, Department of Biology and Biotechnology “C. Darwin”, Sapienza University, Rome, Italy; University of Cape Town, South Africa

## Abstract

The single amino acid replacement Asp116His distinguishes the two subtypes HLA-B*2705 and HLA-B*2709 which are, respectively, associated and non-associated with Ankylosing Spondylitis, an autoimmune chronic inflammatory disease. The reason for this differential association is so far poorly understood and might be related to subtype-specific HLA:peptide conformations as well as to subtype/peptide-dependent dynamical properties on the nanoscale. Here, we combine functional experiments with extensive molecular dynamics simulations to investigate the molecular dynamics and function of the conserved Arg62 of the α1-helix for both B27 subtypes in complex with the self-peptides pVIPR (RRKWRRWHL) and TIS (RRLPIFSRL), and the viral peptides pLMP2 (RRRWRRLTV) and NPflu (SRYWAIRTR). Simulations of HLA:peptide systems suggest that peptide-stabilizing interactions of the Arg62 residue observed in crystal structures are metastable for both B27 subtypes under physiological conditions, rendering this arginine solvent-exposed and, probably, a key residue for TCR interaction more than peptide-binding. This view is supported by functional experiments with conservative (R62K) and non-conservative (R62A) B*2705 and B*2709 mutants that showed an overall reduction in their capability to present peptides to CD8+ T cells. Moreover, major subtype-dependent differences in the peptide recognition suggest distinct TCR binding modes for the B*2705 versus the B*2709 subtype.

## Introduction

**Figure 1:**
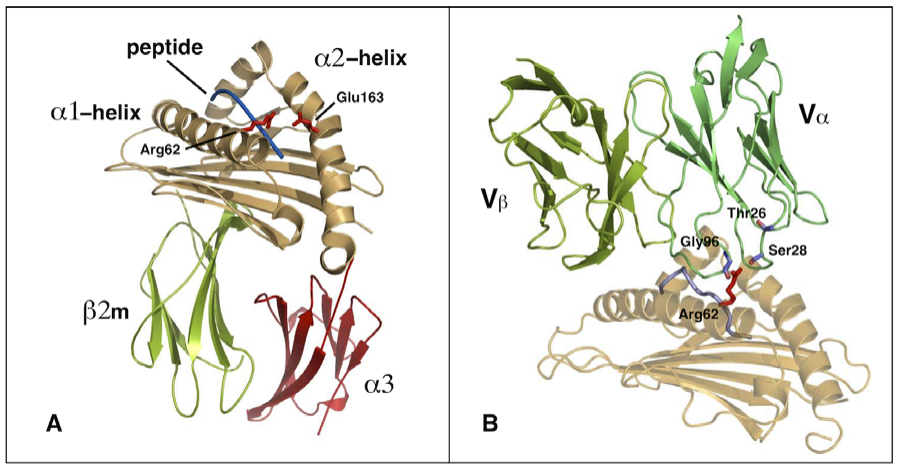
MHC:peptide complex unbound (A) and bound (B) to a TCR. A) Cartoon representation of HLA*B2705 presenting the pLMP2 peptide (PDB ID: 1UXS). The peptide-binding groove is shown in beige, the α3 domain in red, the β2-microglobulin in green and the bound LMP2 peptide in blue. Arg62 and Glu163 are emphasized in stick representation. B) Cartoon representation of the binding groove of HLA-B*0801 (in beige) in complex with an EBV derived nonapeptide (in purple) bound to the Vα and Vβ chains of the LC13 T-Cell receptor (in green, PDB ID: 1MI5). Arg62 of the MHC binding groove as well as the residues of the Vα chain making contacts with Arg62 (Thr26, Ser28 and Gly96) are shown in stick representation.

Major histocompatibility complex (MHC) class I molecules are highly polymorphic glycoproteins involved in the presentation of foreign peptides to cytotoxic CD8+ T lymphocytes. In this process, which is pivotal for immune surveillance of intracellular pathogens, a key step is the recognition of the MHC class I complex (heavy chain (HC), β_2_-microglobulin (β_2_m) and peptide, see Fig. 1A) by a T-cell receptor (TCR). Peptides, typically 8–10 amino acid residues long, are produced during intracellular protein degradation [1]. A detailed knowledge of the rules governing peptide binding arose from crystallographic studies and peptide elution analysis from purified MHC molecules [2]–[7]. Less is known about the dynamic properties of the MHC class I complex [8] and whether or how the peptide or the heavy chain binding groove may adapt their conformation in the process of recognition by TCR, either by induced fit or by conformational selection [9]–[12].

HLA-B27 is one of the most investigated human MHC class I antigens given the strong association with Ankylosing Spondylitis (AS), a rheumatic autoimmune disorder [13], [14]. Nevertheless, HLA-B27 confers to carriers some immunological benefits such as effective cytotoxic T cell (CTL) responses by presenting epitopes from many infectious agents such as influenza virus (flu), Epstein-Barr virus (EBV), hepatitis C virus (HCV) and human immunodeficiency virus (HIV) [15]–[18].

The pathogenic role of HLA-B27 has not yet been fully elucidated. Notably, some HLA-B27 subtypes are not associated with AS [19]–[21]. This applies to the HLA-B*2709 allele which occurs in up to 19% of B27 healthy carriers in Sardinia [22]. B*2709 represents a good investigative tool in pairwise comparative studies with B*2705, the most common B27 allele and strongly associated with AS in worldwide populations. Indeed, the two allelic products are distinguished only by a single substitution in the residue 116 (Asp in B*2705 and His in B*2709) located in the floor of pocket F where the peptide C-terminus accommodates [23]. Asp116His is a relevant polymorphism that gives rise to different repertoires of bound peptides and cytotoxic CD8+ T cells (CTL) [24]–[26]. As an example, pVIPR, a self-peptide derived from type I receptor of Vasoactive Intestinal Peptide evokes autoreactive CTL responses in B*2705 individuals, mostly patients with AS, but not in B*2709 healthy individuals [26], [27]. This peptide exhibits a double conformation, canonical (pVIPR A) and non-canonical (pVIPR B) on B*2705, and only the canonical (pVIPR A) binding mode in complex with B*2709 [28]. This finding allows speculating on a cause-effect correlation between the double conformation of pVIPR and a defective negative thymic selection that prevents autoreactive CTLs to be deleted thus allowing them to gain access to the circulating T cell pool. pVIPR shares high sequence similarity with pLMP2, a viral peptide from EBV which is displayed in two drastically different conformations by B*2705 (non-canonical) and B*2709 molecules (canonical) [29]. The remarkable structural similarities between pLMP2 and pVIPR on B*2705 molecules are functionally mirrored by the occurrence of pLMP2/pVIPR cross-reactive CTL in B*2705 positive patients with AS [29]. Both these peptides have an Arg at position 1, a feature shared by a large portion of B27 bound peptides [30]. Crystallographic analysis revealed tight interactions of this residue bound in the A pocket to the three residues Glu163 (α2-helix), Trp167 (α2-helix), and Arg62 (α1-helix). Both van der Waals interactions as well as water-mediated salt bridges between these residues contribute significantly to the peptide binding [28], [29], [31], [32].

Until now, no crystal structure of TCRs bound to B27:peptide complexes has been solved. Therefore, no information on conformational changes in these structures upon TCR engagement is available. However, several crystal structures of other MHC complexes bound to TCRs and sharing common key residues (Arg62, Gln65, Ala150) with the two B27 subtypes investigated here are available in the Protein Data Bank [33]–[35]. Notably, in all these structures and independently from the initial state, the Arg62 side chain adopts a conformation enabling the contact with the CDRα1 once the TCR engages the MHC:peptide (Fig. 1B). In the pLMP2:, pVIPR: and TIS:B*2705/09 crystal structures, the Arg62 side chain is always oriented towards the HLA binding groove and even engaged in a (water-mediated) salt bridge to Glu163 of the opposed α2-helix [28], [29], [32]. The salt bridge is formed across the N-terminal part of the peptide. In this conformation, the Arg62 side chain is hardly accessible for interactions to a TCR (see Fig. 1A).

The time evolution of conformations sampled by a biomolecular system (here MHC:peptide complexes) can be monitored at atomic resolution by performing molecular dynamics (MD) simulations [36]. MD simulations were already applied to the study of various HLA-B molecules in complex with different peptides [8], [37]–[43].

In the present study, MD simulations of B*2705 and B*2709 molecules bound to peptides having an Arg (pVIPR, pLMP2 and TIS) [44] or a Ser (NPflu) [45] at position 1, document a TCR-independent but peptide-dependent conformational change of Arg62. The simulations show a predominant solvent-exposed conformation of the Arg62 side chain for B27:peptide complexes with an arginine in position 1 of the peptide.

The relevance of Arg62 for T-cell recognition was investigated by functional experiments in which self (pVIPR) or viral peptides (pLMP2, NPflu) are presented to specific CTLs in association with B*2705 or B*2709 mutants in which a Lys or an Ala replaces Arg62.

## Results

### MD simulations of HLA-B27:peptide systems

Crystal structures of B*2705 and B*2709 with bound peptides were resolved at 100 K [28], [29], [32]. Here, we investigated the conformational dynamics focusing on the Arg62 residue of seven different HLA:peptide systems at physiological temperature, by MD simulations of 400 ns length. The simulated systems were pLMP2, pVIPR, TIS associated with either B*2705 or B*2709 molecules, and NPflu in complex with B*2705 only (see Methods). TIS is part of TIS11B, a member of epidermal growth factor early response genes that has been eluted from both B*2705 and 09 molecules [24], [46], [47]. Thermodynamic and structural studies have demonstrated an almost total equivalence of B*2705:TIS and B*2709:TIS complexes [32], [44] and therefore this peptide has been included in the MD simulations as reference. NPflu is an immunodominant, HLA-B27-restricted epitope derived from the nucleoprotein of Influenza A virus and the only investigated peptide here lacking Arg at P1 [45]. No simulation of B*2709:NPflu was undertaken given the instability of this complex caused by the presence of pArg9 (C-terminal anchor) which is disfavoured in the F pocket of B*2709 (His 116) [24], [47].

### Arg62 conformational flexibility in B*2705 and B*2709 subtypes is peptide-dependent

**Figure 2:**
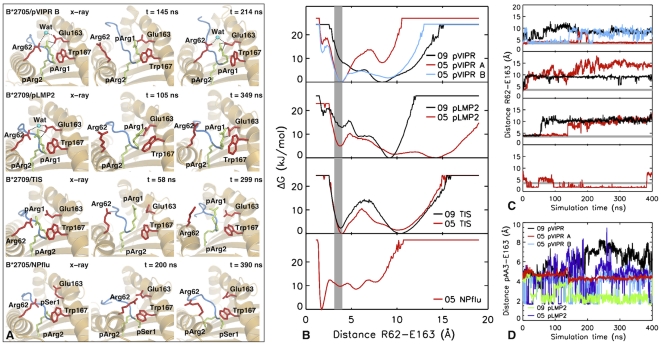
Conformational variability of the Arg62 side chain. A) Representation of the N-terminal binding pockets of HLA-B*2705 presenting pVIPR in non-canonical conformation (top row), of HLA-B*2709 presenting pLMP2 (2nd row) and TIS (3rd row), and of HLA-B*2705 presenting the NPflu peptide (bottom row). Residues of the binding groove (Arg62, Glu163 and Trp167, red sticks) and the first two N-terminal residues (pArg1 and pArg2) of the peptide (in blue) are highlighted. The x-ray structures of the respective systems are shown in the first column. For comparison, three representative snapshots taken at different times of the simulations are depicted. Water molecules involved in water mediated salt-bridge between Arg62 and Glu163 are shown if present (blue sphere). B) Free energy profiles as a function of the distance between Arg62 and Glu163 computed for all eight simulated systems. The gray area indicates the range of the distances found in the corresponding crystal structures. C) Time traces of the distance between Arg62 and Glu163 as obtained from the MD simulations (legends as in B). D) Time traces of the distance between Glu163 and pArg3 of pLMP2 and pLys3 of pVIPR, respectively.

In pLMP2 and pVIPR pArg1 forms a direct salt bridge with Glu163 in both B*2705 and B*2709 subtypes as shown by the x-ray crystal structures (left panels in Fig. 2A). As a measure of the Arg62 conformations sampled in the simulations, the minimum distance between Arg62 and Glu163 (see Fig. 1A) was calculated as a function of the simulation time for all systems. The distribution of distances was used to analyze the free energy profile along this degree of freedom, (ΔG(dist)) [48]:
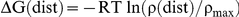
ρ(dist) is the density of states at a given distance and ρ_max_ the maximal density of the respective distribution [48]. The ΔG profile as well as the time traces for the distance between Arg62 and Glu163 are shown in Figure 2B and C. The grey area indicates the distance range as observed in the crystal.

The side chain of Arg62 showed a large conformational flexibility for all systems involving peptides with a N-terminal Arg. In these systems, the water-mediated salt bridge between Arg62 and Glu163 present in the crystal structures was destabilized resulting in a global rearrangement of the A pocket. As shown in Figure 2A, the side chain of Arg62 adopted a completely solvent exposed conformation. This exposed conformation resembles the one observed for the same side chain in the different reported MHC/TCR crystal structures (see Fig.1B) [34]. A local energy minimum corresponding to the bound conformation observed in crystal structures was found for both B*2705 and B*2709 subtypes presenting peptides with Arg in position 1 (pVIPR, pLMP2, TIS). In these systems, the free energy profile additionally shows the presence of stable exposed conformations of the Arg62 side chain (local/global energy minimum corresponding to Arg62-Glu163 distances >6.0 Å). For B*2705 in complex with pLMP2, distances between Arg62 and Glu163 of 15 Å and more were observed (Fig. 2B). This separation is due to a partial unfolding of the α1-helix resulting in an increased separation between the two helices [43]. The contact between Arg62 and Glu163 present in the starting structure and lost during the MD simulation is intermediately restored for HLA-B*2705 in complex with pVIPR in both A and B conformations (Fig. 2A top panel and 2C).

The breakage of the water-mediated salt-bridge between Arg62 and Glu163 is facilitated by the positive charges of the guanidinium group of pArg1 and of pArg3/pLys3. These can partially stabilize the negative charge of Glu163 after loss of contact with Arg62 by formation of transient, partially water-mediated, salt bridges to Glu163 (see Fig. 2D for distances between pArg3/pLys3 to Glu163). Additionally, the Glu163-Arg62 interaction may be replaced by a salt bridge to the positively charged N-terminal amine group instead of the guanidinium group of pArg1. This occurs in the B*2705:pLMP2, B*2709:pLMP2 and B*2709:pVIPR complexes.

Intriguingly, the only investigated system B*2705:NPflu with a peptide lacking pArg1 but having pSer1, an uncharged, albeit polar residue as well as pTyr3, showed a considerably different energetic profile when compared with the other systems. A significantly more stable salt bridge (not water-mediated) between Arg62 and Glu163 was observed in the simulations. Such an interaction is absent in the crystal structure of NPflu [45].

### R62K and R62A replacements in B*2705 and B*2709 subtypes do not alter the cell surface expression and molecular stability

**Figure 3:**
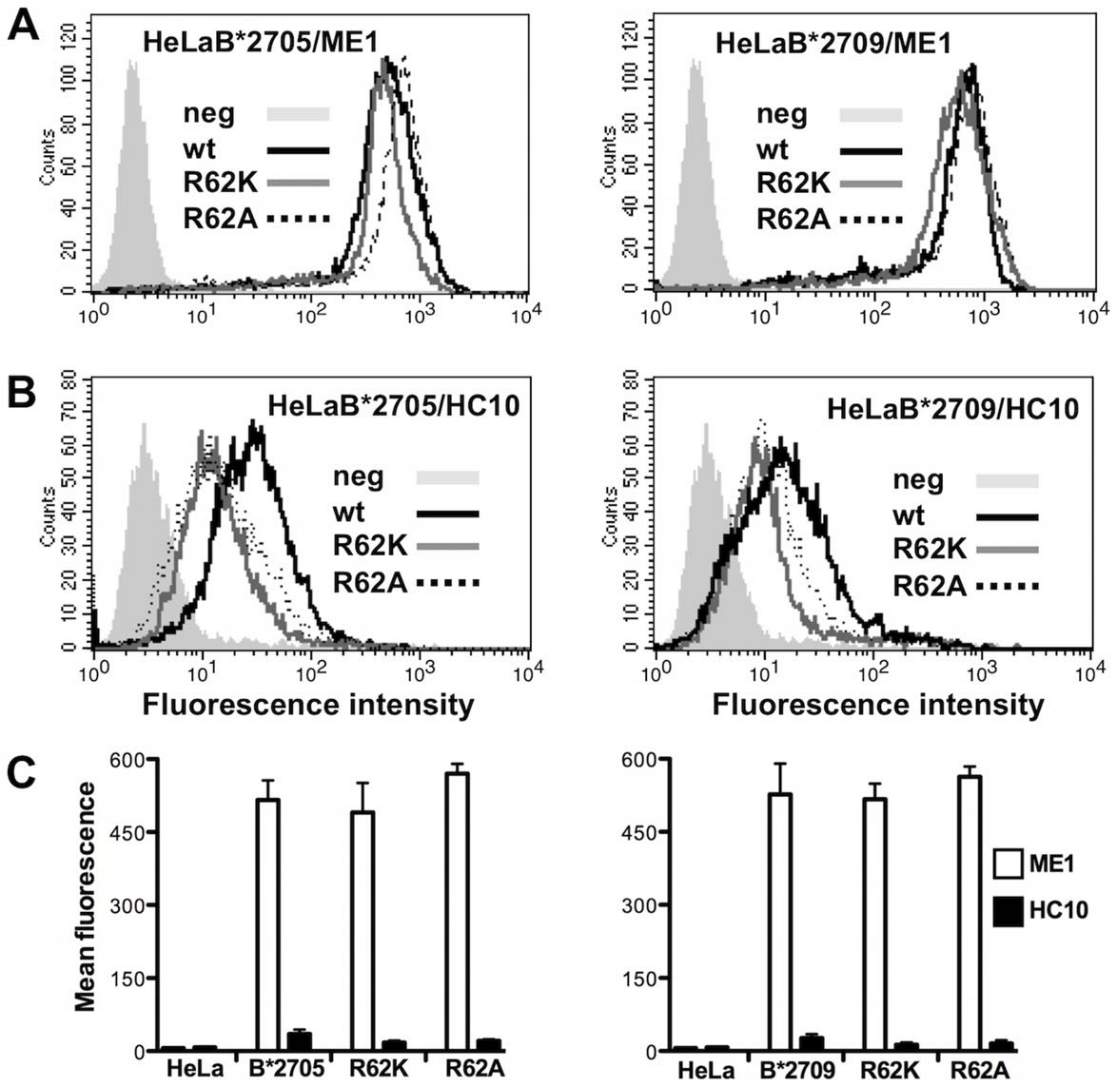
Cell surface expression of R62A and R62K B*2705/09 mutants on Hela transfectants. A) Surface expression of R62A and R62K mutants within B*2705 (left panel) and B*2709 context (right panel) compared to that of wt molecules. Cells were stained with ME1 mAb and analysed by flow cytometry analysis. B) Surface expression of free heavy chains of R62A and R62K B*2705 mutants (left panel) and R62A and R62K B*2709 mutants (left panel) versus wt molecules analysed with HC10 mAb. Grey histograms represent wt HeLa cells (neg) used as controls. Mouse IgG negative controls have not been shown. One representative experiment is shown here. C) Comparison of the surface expression of wt (B*2705 on left panel; B*2709 on right panel) molecules with the relative mutants as folded heterodimers (white bars) and β2m-free heavy chains (black bars). Untransfected HeLa cells (HeLa in the figure) do not expressed unfolded heavy chains. The results are expressed as mean fluorescence ± SD of five/six independent experiments.

In order to investigate the functional role of Arg62, we performed single amino acid substitutions in both B27 subtypes. In particular, we generated B*2705 and B*2709 mutants substituting Arg62 by Lys (R62K) that conserves the positive charge and by Ala (R62A) in which a non-polar residue replaces the positively charged residue. For functional experiments, we stably transfected the cDNAs encoding the four B27 mutants in HeLa cells. Afterwards, HeLa transfectants have been cloned and several different clones analysed by flow cytometry to assess the expression of B27 mutants on the cell surface. One clone for each B27 mutant was chosen for further experiments. The B27 mutants expression profile has been analysed with either ME1, a mAb recognizing the properly folded B27 molecules (heavy chain/β2m/peptide) or HC10 that reacts with the heavy chains dissociated from the β2m [49], [50]. The staining with the conformational dependent antibody ME1 showed an expression level of the folded R62K and R62A B27 mutants slightly lower and higher, respectively, than wt (Fig. 3A and C). On the opposite, the analysis with the HC10 mAb showed that both R62K and R62A mutants in the context of B*2709 and, even more, in that of B*2705 displayed an evident decreased amount of β2m-free heavy chains compared to wt molecules (Fig. 3B and C). The data suggest that these mutations do not induce dissociation of the three party complexes on the cell surface. No free heavy chains are detectable on the cell surface of untransfected HeLa cells (Fig. 3B).

### Arg 62 is relevant for TCR recognition of peptides with pArg1 associated with B*2705 and B*2709 molecules

**Figure 4:**
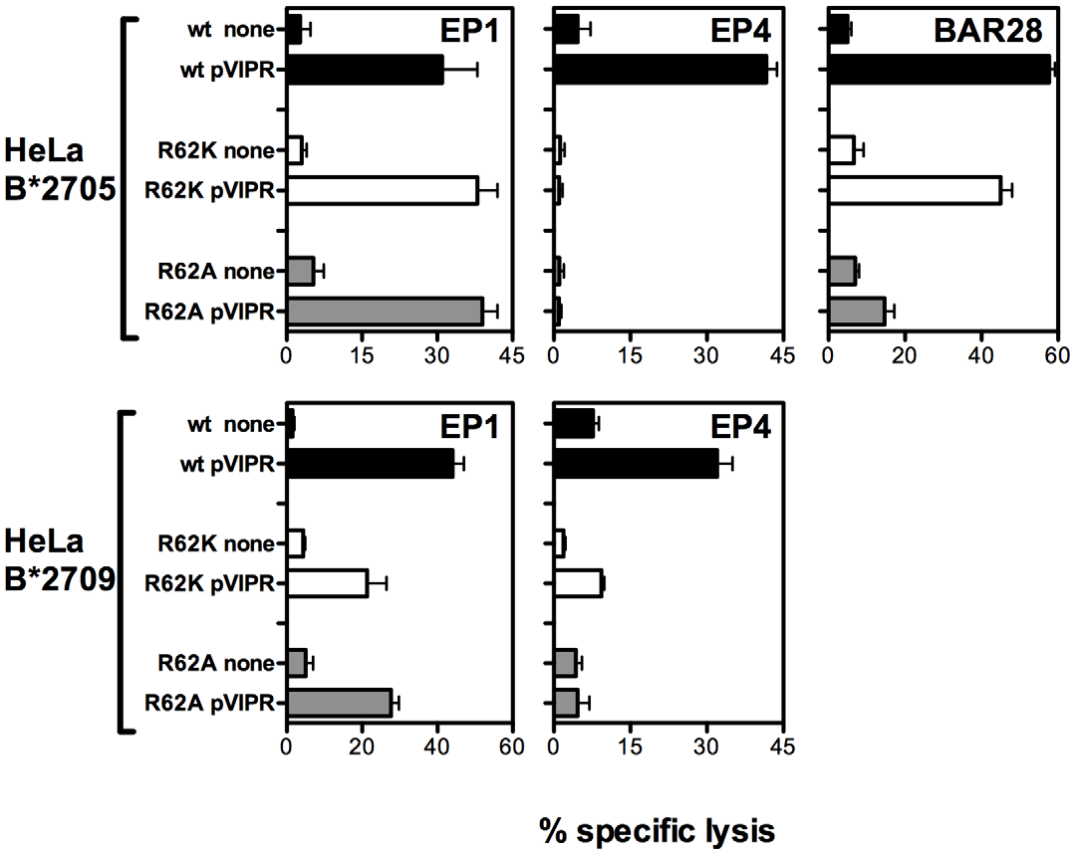
CTL recognition of pVIPR displayed by R62K and R62A B*2705/09 mutants and by wt subtypes. 4 h standard ^51^chromium-release assay showing the reactivity pattern of pVIPR responsive CTLs raised in B*2705 positive patients with AS (EP and BAR) against HeLa cells transfected with B*2705 and 09 molecules or expressing the indicated mutant forms. These cells used as targets have been pulsed ON with pVIPR (70 µM) or cultured in medium alone. Effector/target ratio was 15∶1. Bars represent the mean percentage of lysis ± SD of three independent experiments.

HeLa transfectants expressing the B*2705 and B*2709 mutant forms have been used as target cells for specific HLA-B27-restricted CD8+ cytotoxic T cells to assess whether the substitution of Arg62 by a conservative (Lys) or non-conservative (Ala) amino acid could affect the T cell recognition of pVIPR and pLMP2. The results shown in Figure 4 attest a distinct behaviour among three different pVIPR-specific CTL lines derived from two B*2705 positive patients with AS (EP and BAR). In the case of EP1 CTL line, the pVIPR:B*2705 mutant complexes were recognized even better as compared to the wt molecules. On the contrary, the same CTL line was less effective when the peptide was presented by either R62K or R62A B*2709 mutants compared to the wt molecules. As for EP4 from the same patient with AS, the TCR recognition appeared highly dependent on the presence of Arg62 since the recognition of pVIPR was totally abrogated on both R62K and R62A B*2705 mutants and on R62A B*2709 mutant and heavily reduced (up to 70% on R62K B*2709 mutant (Fig. 4).

The effector functions of BAR28 CTL line were tested only in the B*2705 context. It exhibited a clear lack of peptide recognition on the non-conservative mutant R62A, and a partial decrement on the R62K mutant compared to the wt (Fig. 4).

**Figure 5:**
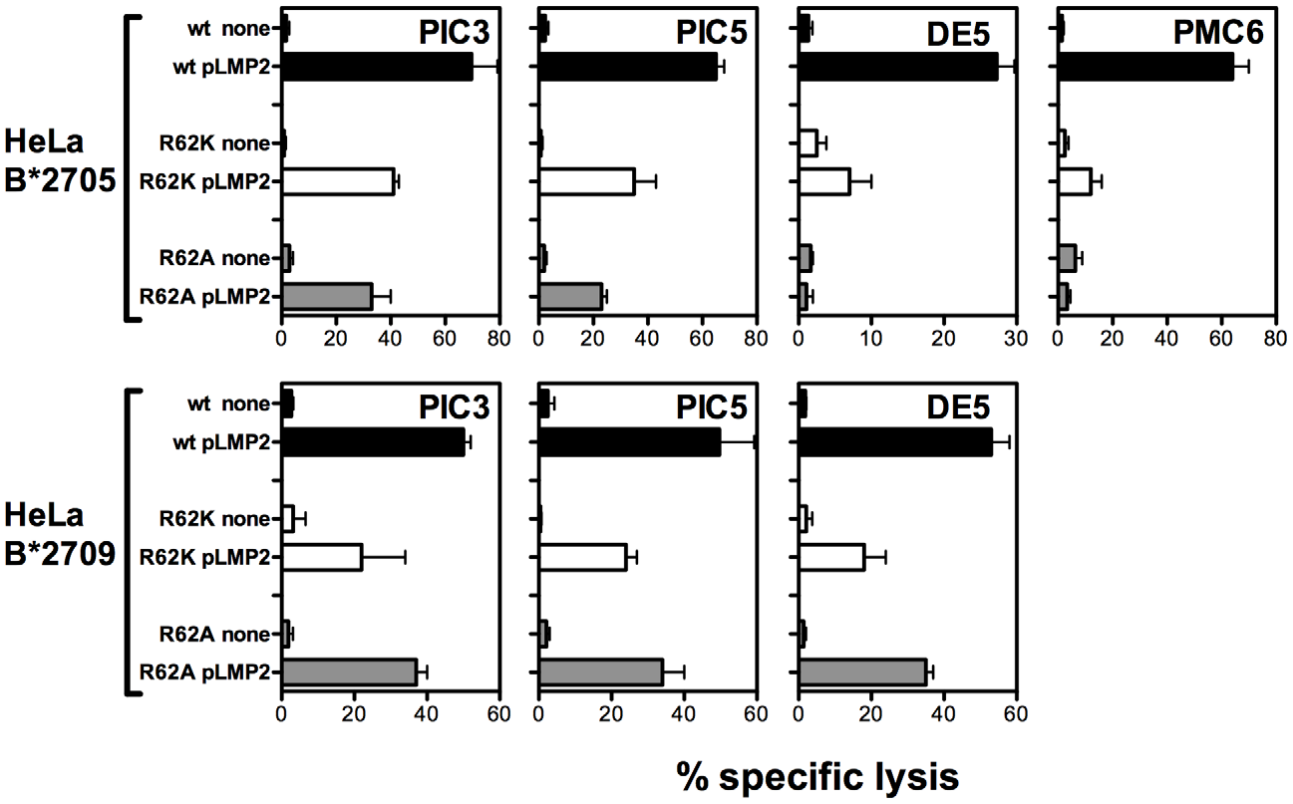
CTL reactivity against pLMP2 bound to R62K and R62A B*2705/09 mutants and wt molecules. pLMP2-driven CTL lines derived from a B*2705 positive patients with AS (PIC) and two B*2709 positive individual (DE and PMC) have been tested in a 4 h standard ^51^chromium-release assay using HeLa transfectants expressing wt or mutants of B*2705 and B*2709 subtypes as target cells after ON treatment with pLMP2 (70 µM) or in absence of peptide. Effector/target ratio was 15∶1. Mean percentage of lysis ± SD of three separate experiments is shown here.

As for the recognition of pLMP2, we tested four CTL lines: two from a B*2705 patient with AS (PIC) and two from two different B*2709 individuals (DE and PMC). PIC3 and PIC5 CTL lines behaved similarly showing reduced lytic activity against both B*2705 and B*2709 mutant HeLa cells incubated with pLMP2 in comparison with the wt molecules (Fig. 5). Interestingly, pLMP2 was recognized slightly better when presented by the conservative mutant in B*2705 context vs the non-conservative one, while the opposite occurred in the B*2709 context. DE5 displayed only a negligible recognition of pLMP2 on mutant R62K B*2705 and the same trend of PIC3 and PIC5 with the B*2709 mutants. The CTL line PMC6 tested only in the B*2705 context, showed lack of reactivity towards pLMP2 on both mutants. No CTL line, either pVIPR- or pLMP2- specific, was able to lyse untransfected HeLa cells incubated with the relevant peptide (data not shown).

These results demonstrate that the Arg62, located in the A-pocket of HLA-B27 binding groove, could play an important role as TCR docking residue.

### Arg62 is crucial for TCR recognition also when the peptide possesses a p1 residue different from Arg

**Figure 6:**
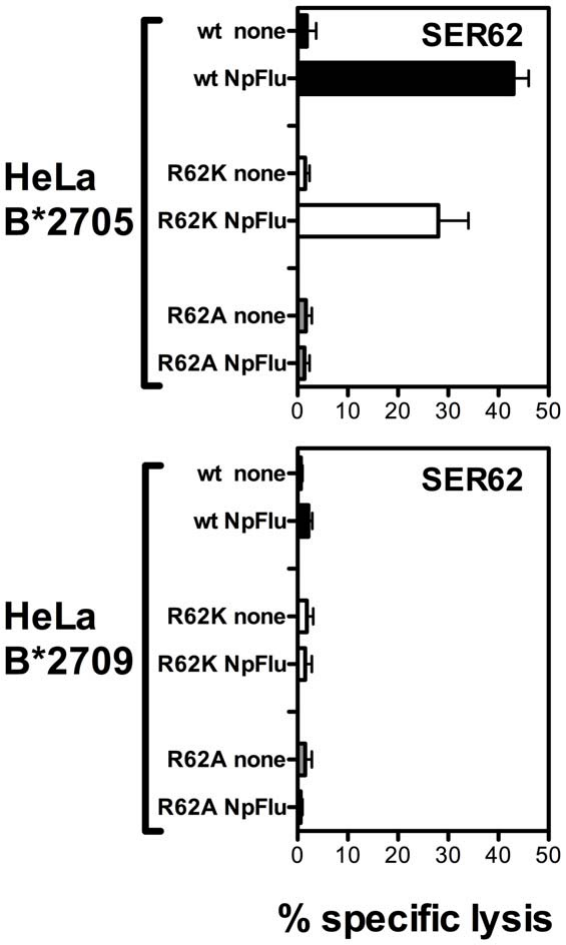
Recognition of Npflu associated to R62K and R62A B*2705/09 mutants and wt molecules. Cytotoxic activity of a specific CTL obtained from a B*2705 positive subject (SER) against HeLa cells transfected with wt and B*2705 and B*2709 mutants and incubated with NPflu peptide (70 µM) or in medium alone and used as targets. Effector/target ratio was 15∶1. Bars represent the mean percentage of lysis ± SD of three independent experiments.

The effect of Arg62 substitutions in the B27 molecules was also investigated with a peptide not having Arg at position 1. In this case, the constellation among Arg62, Glu163 and Trp167 that stabilizes peptides with pArg1 is different. To address this issue, we tested the recognition of NPflu by a specific CTL line (SER62) derived from a B*2705 positive individual. As expected, the B*2709 molecules were not able to present the peptide because of the Arg at the C-terminus (Fig. 6). In the B*2705 context, the non-conservative substitution R62A abrogated the peptide recognition while R62K mutant showed a 35% reduction of lytic activity compared to the wt molecules. This result obtained with a B27 epitope having Ser instead of Arg at P1, suggests a general relevance of Arg62 for TCR recognition of B27/peptide complexes that is only partially influenced by the amino acid specificities at P1 residue.

## Discussion

Subtype- and peptide-dependent structural and dynamical features of the HLA:peptide complexes dictate the conditions for T-cell recognition and are likely to be involved in the differential association of some HLA-B27 subtypes with AS. To investigate this matter, a combination of theoretical and experimental procedures was employed.

We focused on the interactions established by the Arg62 inside the A pocket that anchors the peptide N-terminus. This is a residue of the α1-helix, highly conserved in the alleles of the HLA-B locus (>95%) whose orientation could be strongly influenced by the P1 residue of the specific ligand [51], [52]. In all resolved B27 crystal structures, this arginine is oriented towards the binding groove, frequently forming water-mediated salt bridges to Glu163 on the opposed α2-helix, thereby clamping the peptide in the binding groove. In contrast, available MHC:peptide:TCR structures suggest an engagement of Arg62 in TCR binding implying an exposed Arg62 conformation. Examples are given by the allogeneic H-2K^b^ MHC class I molecule bound to an octapeptide and the KB5-C20 TCR (PDB ID: 1KJ2 2.71 Å) [33]; the HLA-B*0801 in association with a nonamer from EBNA3A of EBV bound to the LC13 TCR (PDB ID: 1MI5 2.50 Å) (Fig. 1B) [34] and the HLA-B*3501 in complex with the endecapeptide EPLP bound to ELS4 TCR (PDB ID: 2NX5 2.70 Å) [35]. In the HLA-B*3501 and HLA-B*0801 structures, four key aminoacids Arg62, Gln65, Ala150, Gln155 are engaged by specific residues of the respective bound TCRs. All these residues are shared by B*2705 and B*2709 subtypes. The same residues, with the exception of Gln155 replaced by Arg155, are involved in the contacts between H-2K^b^ subtype and the KB5-C20 T-Cell receptor.

Accordingly, Webb and colleagues [53] predicted that the Arg62 in the H2-Kb e H2-Kbm8 would contact the CDR1α of the anti-HSV T lymphocytes and, serving as “electrostatic guide for TCR docking”, would be pivotal for T-cell selection depending on its dynamics and positioning.

The tight Arg62-pArg1-Trp167 stacking interaction has been invoked as a possible explanation for the relative high frequency, in the B27 repertoire, of peptides with dibasic N-terminal motif pArg1-pArg2 [30], that, however, have an intrinsic resistance to cytosolic degradation. To this regard, it has been shown that mutations of Glu163 alone or both Glu163 and Trp167 had a limited effect on the B*2705 molecules for usage of peptides with basic P1 residues, thus indicating that the increased cytosolic stability is responsible for such a preferential binding [54]. However, B*2705 mutants substituted at Arg62 residue have never been generated and assessed for their peptide binding repertoire.

Here, we have investigated the structure and dynamics of the Arg62-Glu163 peptide clamp pair in MD simulations of the HLA-B*2705/09 subtypes bound to peptides with arginine (pVIPR, pLMP2, TIS) and serine (NPflu) at the N-terminal peptide position, under physiological conditions. In our computational setting, all MD simulations of B*2705 and B*2709 subtypes bound to peptides with pArg1 (pVIPR, pLMP2 and TIS) revealed a very large conformational flexibility of the Arg62 side chain. Notably, during the 400 ns simulation, the contact between Arg62 and Glu163 present in the respective crystal structures was lost (examples are shown in Figure 2A) and the side chain of Arg62 adopts a flexible exposed conformation, similar to that displayed in the MHC-peptide/TCR complexes reported above (see also Fig. 1B). This conformational change with the exposed Arg62 was stable in the majority of simulated systems except for B*2705 bound to pVIPR both in the canonical and non-canonical conformations. Here, the Arg62-Glu163 bond is intermediately re-established. The Arg62 reorientation is accompanied by a partial rearrangement of the A pocket. The Arg62 re-orientation was favourable when Arg1 could interact with Glu163 or when other positively charged residues such as pArg3 in pLMP2 or pLys3 in pVIPR could form a transient or stable salt bridge with Glu163. In contrast, with a polar residue present at P1 as Ser in NPflu, the system showed a marked different energetic profile with a tightened Arg62-Glu163 clamp across the peptide, although not present in the crystal [45]. Thus, the peptide sequence dictates the conformation of TCR-accessible heavy chain amino acids. Several MHC:peptide:TCR crystal structures reported in the literature as well as our molecular dynamics simulations suggested a key role for Arg62. For a deeper comprehension of its relevance to the B*2705 and B*2709 molecular stability and, more importantly, to the antigen presentation to TCR, we generated HeLa cells stably expressing R62A and R62K B27 mutant forms. Both conservative and non-conservative substitutions did not significantly change the cell surface expression level of B27 mutants as inferred by comparison with wt B*2705 and B*2709 alleles (Fig. 3). This was observed by flow cytometry analysis with ME1 [49], a mAb recognizing the properly folded B27 molecules or with HC10 [50] that reacts against the free heavy chains. Therefore, a change in the expression level and/or molecular stability of the mutants did not bias T-cell data.

A comparison of T-cell results for pVIPR and pLMP2 presented by the wt molecules and by the R62A and R62K B27 mutants suggests a relevance of R62 for TCR interaction. For all but one CTL line (EP1), the mutations in Arg62 led to a significant decrease or even total abrogation of lytic activity with respect to pVIPR presentation by wt B27 molecules. Interestingly, the EP1 CTL line displayed a full activation by R62K and R62A B*2705 mutants but not by the same mutants in the B*2709 context. We do not expect that mutations of Arg62 lead to a different distribution of pVIPR conformations observed in crystal structures in complex with B*2705 and thereby to a modified T-cell response. The crystal structures of both conformations show a similar interaction pattern in this region of the binding groove.

For pLMP2 presentation in the disease-associated B*2705 context, both mutants are less effective than wt in activating the CTL lytic activity with the non-conservative R62A mutant working slightly worse than the conservative R62K mutant. This order is reversed in the case of peptide presentation by the non-disease associated B*2709 (Fig. 5) suggesting different TCR binding modes to B*2709 as compared to B*2705. DE5 CTL provides a striking example: it failed to react against pLMP2 when presented by R62A B*2705 mutant while it was activated by the same mutant in the B*2709 context. All together, these data strongly suggest that R62 mutations in the A pocket could be differently buffered in respect to TCR recognition by the His-Asp polymorphism at 116 in the F pocket. Also T cell recognition of NPflu, the only peptide analysed here having Ser at P1, appeared to be influenced by mutations at R62 in the B*2705 context while, as already mentioned, it is not presented by the B*2709 subtype. As for the cases of B*2705 presenting pLMP2 or pVIPR, CTL activity required a positively charged residue at position 62 (R or K). This result is consistent with previous T-cell recognition data showing that the side chain of pSer1 was not directly involved in interactions with the TCR but rather the region around P1 represents a docking site for the TCR. Accordingly, the replacement of pSer1 by amino acids with bulky side chains (R, K or Y) induced a marked reduction of T-cell recognition due to the steric interference with TCR binding [45], [55].

This combined experimental and theoretical study would suggest a differential contribution of Arg62 to the TCR recognition of B*2705/09:peptide complexes. MD simulations of the wt B27:peptide systems revealed the meta-stability of the Arg62-Glu163 peptide clamp frequently observed in crystal structures, and the preference of Arg62 to adopt a solvent-exposed, TCR oriented side-chain conformation. Functional experiments showed that the positive charge of Arg62 is preferred for the TCR recognition of disease-associated B*2705 complexes. Differently, a small amino acid such as Ala is favourable over Lys in the non-disease-associated B*2709 subtype. In conclusion, this study gives a strong indication for a B27 subtype-dependent functional role of Arg 62 in the antigen recognition by TCR.

## Materials and Methods

### Ethics Statement

This study has been approved by the Ethics Committee of the University of Cagliari where blood samples of patients with AS and healthy controls have been collected (365/09/CE). All participants involved in this study gave their written informed consent.

### MD simulations

The starting structures of the HLA-B*2709 and HLA-B*2705 proteins presenting the peptides studied here were taken from the Protein Data Bank (PDB entries: 1UXW: B*2709 with pLMP2 at 1.71 Å [29], 61UXS: B*2705 with pLMP2 at 1.55 Å [29], 1OF2: B*2709 with pVIPR at 2.20 Å [28], 1OGT: B*2705 with pVIPR in canonical (VIPR A) and non-canonical (VIPR B) conformation at 1.47 Å [28], 1W0W: B*2709 with TIS at 2.11 Å [32], 1W0V: B*2705 with TIS at 2.27 Å [32]. Additionally the HLA-B*2705-peptide complex with the immunodominant viral peptide from influenza nucleoprotein (NPflu) NP383–391 was also investigated (PDB ID: 2BST) at 2.10 Å [45].

Protonation states of the titratable groups present in the investigated proteins [56] were chosen according to results of pKa calculations performed on the studied systems using the WHATIF package [57].

The GROMACS software package [58] (version 4.0.4) with the OPLS-AA/L all-atom force field [59] was used to carry out the MD simulations. The proteins were solvated in a dodecahedron box, imposing a minimum distance between the protein and the box of 1.4 nm. Almost 27,000 TIP4 water molecules were added to the different systems [60]. Na^+^ and Cl^−^ ions were added to the systems in order to reproduce the physiological ionic concentration (0.15 M). An excess of Na^+^ was added to compensate for the net negative charge of the MHC complexes. The long-range electrostatic interactions (distances >1.0 nm) were computed by the Particle Mesh Ewald (PME) method [61]. The short-range electrostatic interactions were treated explicitly with a non-bonded pairlist cut off of 1.0 nm. The bond lengths of the hydrogen atoms were constrained by applying the Lincs algorithm thus permitting the use of a time step of 2 fs for numerical integration of the equations of motion [62]. The temperature was kept constant by coupling the system to an external thermal bath (310 K) with a coupling time constant τ_T_ = 0.1 ps [63]. The systems were weakly coupled to a pressure bath (1 Bar) [63] with a coupling time constant τ_p_ = 1.0 ps. 200 steps of energy minimization (steepest descent algorithm), followed by 100 ps of MD simulation with harmonic position restraints (force constant 1000 kJ mol^−1^ nm^−2^) on the heavy atoms of the protein preceded the production runs. All MD simulations were run for 400 ns (2×10^8^ integration steps), adding up to a total simulated time of 3.2 µs.

### Synthetic peptides

The following peptides have been used in this study: pVIPR (RRKWRRWHL, 400–408) from human vasoactive intestinal peptide type 1 receptor [26]; pLMP2 (RRRWRRLTV, 236–244) [16] from Epstein-Barr virus latent membrane protein 2; NPflu (SRYWAIRTR, 383–391) [15], [45] from influenza A virus nucleoprotein; TIS (RRLPIFSRL, 325–333) [24] from TIS11B, member of epidermal growth factor early response genes. Peptides were purchased from PRIMM GmbH (Dubendorf, Zuerich, CH), dissolved in 100% DMSO and their concentration estimated by BCA test according to the manufacturer's protocol (Pierce, Thermo scientific, IL, USA).

### B27 mutants and cDNA cloning

cDNAs encoding for B*2705 and B*2709 had been cloned in pCEP4 mammalian expression vector (Invitrogen, Carlsbad, CA, USA) as reported previously [23]. The mutated constructs were generated by PCR-based cassette mutagenesis of the B*2705 and B*2709 wt cDNAs by using the following primers: (forward) 5′-GCCCGGTACCGGACTCAGAATCTCCTCAG-3′ and (reverse) 5′-CAATACTCCGGACCCTCCTGCTCTATCC-3′ that amplify part of HLA-B27 cDNA upstream to the codon encoding for R62. This 240 bp fragment that included part of 5′-UTR of HLA-B27, introduced a KpnI site (underlined) at 5′-end and a Kpn2I site (underlined) at 3′-end. A second PCR was performed to introduce the specific mutation R62A and R62K using as primers: forward for R62A substitution 5′-GAGGGTCCGGAGTAT TGGGAC**GCG**GAGACAC-3′ (in bold the codon introducing the mutation) and forward for R62K substitution 5′-GAGGGTCCGGAGTATTGGGAC**AAG**GAGACA-3′ (in bold the codon introducing the mutation) and reverse, 5′- CCGCAAGCTTCTGGGGAGGAAACACAGGTCAGCGGAAC-3′ (the same for all mutants). These primers amplified a fragment of 900 bp and introduced a Kpn2I site at 5′-end (underlined) and a HindIII site (underlined) at 3′-end. The PCRs were conducted in a standard buffer with 2.5 mM MgSO_4_, 10 pmol of each primers, 0,2 mM of dNTP mix, 1,25 U of Pfu Taq Polymerase (Fermentas, Thermo scientific, IL, USA) and H_2_O to a final volume of 50 µl. After a first step at 94°C (30 s), annealing at 62°C (30 s), and extension at 72°C (30 s) for 5 cycles, other 27 cycles were run at 94°C (30 s), 66°C (30 s), 72°C (30 s), before a final extension at 72°C (7 min) in a GeneAmp PCR system 9700 Thermal cycler (Applied Biosystems, Carlsbad, CA, USA). The PCR products were sequentially digested with KpnI (Fermentas, Thermo scientific, IL, USA) and Kpn2I (Fermentas, Thermo scientific, IL, USA) as for the fragment of 240 bp and with Kpn2I and HindIII (Fermentas, Thermo scientific, IL, USA) as for the fragment of 900 bp and then purified from agarose gel by using GFX™ PCR DNA and Gel Band Purification Kit (GE Healthcare Europe GmbH, MI, Italy). Afterwards, the 240 bp and the 900 bp fragments of the respective B27 subtypes were mixed at ratio of 5∶5∶1 with pCEP4 vector cut with KpnI and HindIII and gel purified using the same kit as above. The ligations were conducted overnight at 16°C with T4 DNA ligase (New England Biolabs, Beverly, MA). XL1-Blue *Escherichia coli* electrocompetent cells (Stratagene; Agilent Technologies, Italy) were transformed by the products of ligations and several colonies for each subtypes were screened by B27-specific PCR and the positive ones checked by automated DNA sequencing.

### Transfection of HeLa cells

HeLa cells (ATCC number CCL2™; www.atcc.com/) were transfected with pCEP4 constructs containing cDNAs for wt B*2705 and B*2709 and R62A and R62K mutants using Lipofectamine 2000 Reagent (Invitrogen, Carlsbad, CA, USA) according to the manufacturer's instructions. After transfection, cells were diluted in DMEM supplemented with 10% heat-inactivated fetal bovine serum, 2 mM L-glutamine, 100 U/mL penicillin and 100 µg/mL streptomycin at 1×10^6^ cells/mL. Selection for hygromycin B-resistant cells was initiated 48 h post-transfection with 200 µg/mL hygromycin B (PAA, Pasching, Austria). After two weeks of culture, cells were diluted at one cell/well in 96-well flat-bottom microplates and clones, stably expressing B27 molecules, were selected and used to perform subsequent experiments.

### mAbs and immunofluorescence

For immunofluorescence experiments, cells were stained with ME1 (a conformational dependent IgG1mAb, recognizing HLA-B27, -B7, -B42, -B67, -B73 and Bw22) [49] and HC10 (a IgG2a mAb, reacting with a determinant on β2-microglobulin free heavy chains of HLA-B, -C and some HLA-A alleles) [50] and then by F(ab')2 of rabbit anti-mouse FITC (Jackson ImmunoResearch Europe Ltd., Suffolk, UK). Isotype matched mouse Igs were used as negative controls to define background staining. Flow cytometry analysis was made immediately after staining, without fixation, by using a FACSCalibur (BD Biosciences). For each sample, 10.000 events were acquired using forward/side light scatter characteristics and analyzed using Cell Quest software (Becton Dickinson).

### Cell lines

Autologous B lymphoblastoid cell lines (B-LCLs) from B27 positive subjects were generated by *in vitro* immortalization of B cells using the standard type 1 Epstein-Barr virus isolate B95.8 [23] and cultured in RPMI (Lonza, Basel Switzerland) supplemented with 10% fetal calf serum, 2 mM L-glutamine, 100 units/ml penicillin, 100 µg/ml streptomycin. wt and transfected HeLa cells, described above, were cultured in DMEM (Lonza, Basel Switzerland) supplemented with 10% fetal bovine serum, 2 mM L-glutamine, 100 units/ml penicillin, 100 µg/ml streptomycin. HeLa transfectants were cultured in presence of 200 µg/ml hygromycin B (PAA, Pasching, Austria) to maintain the expression of B27 molecules.

### Generation of antigen-specific CTL lines

Peripheral blood mononuclear cells from B*2705 positive patients with AS and healthy donors, either B*2705 or B*2709 positive, were isolated by density gradient centrifugation with Lymphoprep and depleted of the CD4+ T cells by Dynabeads M-450 CD4+ (Dynal ASA, Oslo, Norway). Cell cultures were seeded at 1×10^4^ cells/well in 96-well flat-bottom microplates and stimulated by autologous B-LCLs at 0.5∶1 antigen-presenting cells/responder ratio. The antigen-presenting cells had been pulsed overnight with pVIPR (70 µM), pLMP2 (50 µM) or NPflu (50 µM) peptides before being γ-irradiated (200 Gy). CTL lines were grown in RPMI 1640 medium supplemented with 10% heat-inactivated pooled human serum, 2 mM L-glutamine, 100 U/mL penicillin and 100 µg/mL streptomycin. 20 units/ml human rIL-2 (Roche Applied Science) was added to each well after 3 days. CTL lines were then re-stimulated on day 10–12. One week later, the specificity of CTL lines was tested by a standard ^51^Cr release assay using as targets peptide-pulsed autologous B-LCL and T2B*2705 transfectants [64]. Phenotypic analysis of peptide-specific CTL lines was performed by immunostaining using the following monoclonal antibodies: OKT3, OKT4, and OKT8 (Orthodiagnostics, Stanford, CA). CTL lines were maintained in culture by weekly stimulation with γ-irradiated autologous B-LCL in complete RPMI medium (see above) and human rIL-2 (20–100 units/ml), and were used for functional assays 8–10 days after stimulation. The study has been approved by the Institutional Review Board of the University of Cagliari were blood samples have been collected.

### ^51^Cr-Release Assay

Specific reactivity of CTL lines towards pVIPR, pLMP2 and NPflu was tested by a standard 4-h ^51^Cr-release assay. Target cells (HeLa transfectants either expressing wt B*2705 and B*2709 molecules or R62A and R62K mutated forms) were incubated overnight with the different peptides at indicated concentrations or cultured in medium alone. One day later, target cells were labeled with sodium ^51^chromate, washed and plated (3×10^3^ target cells/well) with effector T cells at 15∶1 effector/target ratio, in absence of free peptide.
